# Neural mechanisms of subclinical depressive symptoms in women: a pilot functional brain imaging study

**DOI:** 10.1186/1471-244X-12-152

**Published:** 2012-09-21

**Authors:** Jennifer N Felder, Moria J Smoski, Rachel V Kozink, Brett Froeliger, Joseph McClernon, Joshua Bizzell, Christopher Petty, Gabriel S Dichter

**Affiliations:** 1 Carolina Institute for Developmental Disabilities, University of North Carolina at Chapel Hill School of Medicine, CB# 3366, 101 Manning Drive, Chapel Hill, NC, 27599-7160, USA; 2 Department of Psychiatry and Behavioral Sciences, Duke University Medical Center, Durham, NC, USA; 3 Department of Psychiatry, University of North Carolina at Chapel Hill School of Medicine, Chapel Hill, NC, USA; 4 Duke-UNC Brain Imaging and Analysis Center, Duke University Medical Center, Durham, NC, USA; 5 Department of Psychology and Neuroscience, University of Colorado Boulder, UCB 345, Boulder, CO, 80309-0345, USA

**Keywords:** FMRI, Depression symptoms, Emotion regulation, Resting state, Reward

## Abstract

**Background:**

Studies of individuals who do not meet criteria for major depressive disorder (MDD) but with subclinical levels of depressive symptoms may aid in the identification of neurofunctional abnormalities that possibly precede and predict the development of MDD. The purpose of this study was to evaluate relations between subclinical levels of depressive symptoms and neural activation patterns during tasks previously shown to differentiate individuals with and without MDD.

**Methods:**

Functional magnetic resonance imaging (fMRI) was used to assess neural activations during active emotion regulation, a resting state scan, and reward processing. Participants were twelve females with a range of depressive symptoms who did not meet criteria for MDD.

**Results:**

Increased depressive symptom severity predicted (1) decreased left midfrontal gyrus activation during reappraisal of sad stimuli; (2) increased right midfrontal gyrus activation during distraction from sad stimuli; (3) increased functional connectivity between a precuneus seed region and left orbitofrontal cortex during a resting state scan; and (4) increased paracingulate activation during non-win outcomes during a reward-processing task.

**Conclusions:**

These pilot data shed light on relations between subclinical levels of depressive symptoms in the absence of a formal MDD diagnosis and neural activation patterns. Future studies will be needed to test the utility of these activation patterns for predicting MDD onset in at-risk samples.

## Background

Despite a large body of research addressing impaired neural functioning in major depressive disorder (MDD), relatively little is known about the neurofunctional characteristics of individuals without MDD but with subclinical levels of depressive symptoms. Studies of individuals who do not meet criteria for MDD but with subclinical levels of depressive symptoms may yield a number of insights: first, they may suggest potential neurobiologic markers of those at risk for MDD [1,2]; second, they may shed light on hereditary and environmental influences on depressive temperament [3]; and third, they may suggest avenues of inquiry regarding the neurobiology of MDD risk and resilience, and thereby inform treatment and preventative intervention approaches [4,5]. The goal of the present pilot study was to investigate linkages between regional brain activation patterns and subclinical depressive symptoms while participants were engaged in emotion regulation, resting state, and reward processing paradigms that have previously been shown to differentiate MDD and nondepressed samples. Below we outline the rationale for selecting each of these three paradigms.

Deficits in emotion regulation (ER) are thought to be central to the core features of MDD [6]. Human and preclinical studies suggest that the prefrontal cortex influences activity in the limbic system in a top-down manner [7,8], and numerous investigations have demonstrated abnormal recruitment of prefrontal cortical regions while individuals with MDD actively regulate responses to emotional stimuli [9,10]. For example, Johnstone and colleagues [6] reported that when instructed to reappraise negatively valenced images, individuals with MDD demonstrated impaired prefrontal cortical inhibition of limbic regions as well as a divergent pattern of covariation between ventromedial prefrontal cortex and amygdala activity. Similarly, Beauregard and colleagues [11] reported right anterior cingulate and insular cortex hyperactivation while individuals with MDD down-regulated their emotional responses to sad images. Finally, Kanske and colleagues [12] reported anterior cingulate and lateral orbitofrontal cortex hyperactivation during emotion regulation in individuals with remitted MDD , supporting the framework that altered neural mechanisms of emotion regulation may indeed represent not only a state marker of MDD illness, but potentially a trait marker of MDD risk as well.

Second, we investigated resting state functional brain connectivity. A mostly midline network of correlated intrinsic brain activity is active during rest and deactive during goal-directed tasks [13,14]. This network has been called both the “baseline state” and the “default mode network (DMN)” [15,16]. The DMN regulates self-referential activities, including evaluating the salience of internal and external cues, remembering the past, and planning the future [13,17]. Individuals with MDD are characterized by altered patterns of DMN activity during emotion processing tasks as well as at rest. Sheline and colleagues [18] reported increased activity in MDD within DMN regions (i.e., ventromedial prefrontal cortex, anterior cingulate, and lateral parietal and temporal cortices), suggesting that MDD is characterized by a failure to down-regulate DMN activity during emotion processing. Another study by the same research group [19] reported increased intrinsic connectivity in MDD between a precuneus seed region and dorsolateral prefrontal cortex (the so-called “dorsal nexus” for overconnected brain regions in MDD) (see also [16] and [20]). Given the centrality of the DMN for self-referential activities, abnormal DMN activity in MDD has been suggested to reflect excessive self-focus accompanied by a decreased ability to attend to cognitive tasks [19].

Finally, we measured frontostriatal responses during a reward processing task because multiple studies have reported altered neural mechanisms of reward processing in MDD (for a review, see [21]). For example, Smoski and colleagues [22] reported that individuals with MDD demonstrated orbital frontal cortex hyperactivity during reward selection that predicted depressive symptom severity and caudate nucleus hypoactivation during reward anticipation. Pizzagalli and colleagues [23] found that individuals with MDD showed decreased putamen responses during reward anticipation and decreased nucleus accumbens and caudate nucleus responses to monetary gains. Finally, Knutson and colleagues [24] reported increased anterior cingulate activation during reward anticipation in MDD. Taken together, these studies highlight not only that MDD is characterized by altered neural activation patterns during reward processing, but that patterns of brain activation differences in MDD are contingent on the temporal phase of the reward response. For this reason, we used a task that allowed for the dissociation of responses during reward selection, reward anticipation, and reward outcome phases of reward responding.

In summary, the purpose of the present study was to extend the literature addressing altered neural mechanisms of emotion regulation, DMN connectivity, and reward processing in MDD by examining the covariation between levels of depressive symptoms and patterns of brain activation in individuals with subclinical levels of depressive symptoms but without MDD diagnoses. Predictions were informed by the extant MDD literature reviewed above [6,19,22]: we hypothesized relations between subclinical depressive symptom severity and (1) lateral prefrontal cortex activity during ER; (2) precuneus-dorsolateral prefrontal cortex connectivity during the resting state scan; (3) lateral prefrontal cortex activity during reward selection; (4) striatal activity during reward anticipation; and (5) medial prefrontal cortex activity during reward outcomes. Given the higher rates of MDD in women [25] and because gender moderates responses to emotional images [26,27], this pilot study examined only females, a strategy that is consistent with other neuroimaging studies examining MDD [28], depression risk [29] and depressive traits [30].

## Methods

### Participants

Participants consented to protocols approved by the local Human Investigations Committees at both the University of North Carolina Chapel Hill and Duke University Medical Center and were recruited from a database maintained at the Duke-UNC Brain Imaging and Analysis Center and by flyers posted in campus and medical center locations. To identify individuals with moderate but not severe depression symptoms, interested respondents first completed a modified the Beck Depression Inventory-II [BDI, [31] via a secure web survey. Next, potential participants completed an in-person screening session that included the full BDI and administration of the Structured Clinical Interview for DSM-IV, Patient Version, with Psychotic Screen (SCID) [32]. The final sample was recruited based on a stratification strategy to recruit equal numbers of individuals with low (i.e., 0–9), medium (i.e., 10–18), and high (i.e., 19+) BDI scores. Exclusion criteria included: 1) current anxiety, substance abuse, or mood disorder beyond dysthymia (as assessed by the SCID), 2) history of mania, 3) currently taking psychoactive medication, 4) magnetic resonance imaging contraindicated (e.g., metal in body), 5) history of neurological injury or disease, and 6) pregnancy. The final sample was twelve females (mean age: 23.3 ± 4.3; 3 African American; 3 Asian, and six Caucasian) who participated in the scan session.

### Pre-scan emotion regulation training session

Participants completed a training session prior to the scan day at which they were taught strategies to distract themselves and reappraise their interpretations while viewing sad stimuli. For the distract condition, participants were trained to think of something positive that was unrelated to what was depicted in the images, such as a fond memory, family member, or an anticipated event. For the reappraise condition, participants were trained to either give the story suggested in the picture a happy ending or alternate meaning, or to tell themselves that no one close to them was affected by the depicted situation. As a control strategy, participants were instructed to experience the image as they normally would without trying to increase or decrease their emotional response (the “view” condition).

### Scan day procedure

On the day of the scan, participants reviewed task instructions and completed the BDI again. During scanning, participants completed the three paradigms described below in a single scan session. Due to technical problems, three participants returned to complete the emotion regulation task at a separate imaging session, at which time the BDI was readministered. Analyses included only BDI scores obtained on the day that each task was completed. The mean BDI scores in final analyses were 11.33 (SD = 10.21, Range = 0 to 26, Skewness = 0.10) on the day of the resting state and reward processing scans, and 9.92 (SD = 9.02, Range = 0 to 24, Skewness = 0.29) on the day of the ER scan.

### Emotion Regulation (ER) task

**Figure 1:**
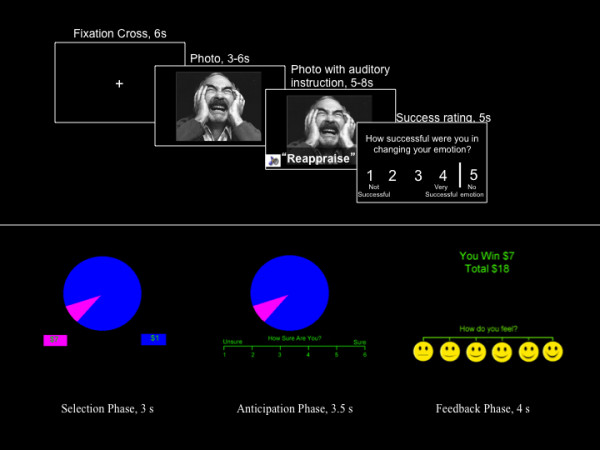
Top: Depiction of the emotion regulation task, showing durations of each phase; Bottom: Depiction of the Wheel of Fortune task; the area of the slices matched the likelihood of winning explicit amounts of money demoted below the wheel in squares of matching colors.

The ER task consisted of four 4-minute 24-second runs with nine sad trials and three neutral trials each (see the top of Figure 1). The stimuli used in the task are described elsewhere [33,34]. During each 22-second trial, participants first fixated for 6 seconds and then saw a sad or neutral image for 11 seconds with an auditory instruction presented 3–6 seconds after image onset. Sad images were presented on 75% of trials and were accompanied by an instruction to “reappraise,” “distract,” or “view” the image. Neutral images, on the other 25% of trials, were accompanied by an instruction to “view” the image. Following image offset, participants reported their ER success on a 4-point scale anchored by “not successful” and “very successful,” or they indicated they felt “no emotion” (trials with “no emotion” were not included in analyses).

### Resting state scan

Participants were instructed to close their eyes and relax but not fall asleep for the duration of the 6-minute resting state scan.

### Wheelof Fortune (WOF) reward processing task

The WOF is described in greater detail elsewhere [35]. Briefly, three 12-minute runs with 46 trials each were composed of selection, anticipation, and outcome phases (see the bottom of Figure 1). During the selection phase (3 seconds), participants viewed a segmented wheel of fortune and indicated their selection. During the anticipation phase (3.5-7.5 seconds), participants rated how sure they were of winning on a scale of 1 to 6 (anchored by “unsure” and “sure”). During the outcome phase (4 seconds), participants viewed the amount won (“$0” for not winning), the total cumulative amount won in the run, and rated how they felt on a pictorial Likert scale. Responses were contrasted with a control condition that lacked monetary stimuli but required active responding.

### Imaging methods

Scanning was performed on a GE 3 Tesla Signa Excite HD scanner with 50-mT/m gradients (General Electric, Waukesha, Wisconsin, USA). Head movement was restricted by foam cushions. An eight-channel head coil was used for parallel imaging. A high resolution T1-weighted anatomical image was acquired using a 3D fast SPGR pulse sequence (68 slices, TR = 500 ms; TE = 20 ms; FOV = 24 cm; image matrix = 256^2^; voxel size = 0.9375 × 0.9375 × 1.9 mm) aligned in the near axial plane defined by the anterior and posterior commissures. Whole brain functional images consisting of 30 slices parallel to the AC-PC plane were collected using a BOLD-sensitive gradient-echo sequence with spiral-in k-space sampling and SENSE encoding to take advantage of the 8-channel coil (TE: 30 ms; FOV: 25.6 cm; isotropic voxel size: 4 mm^3^; SENSE factor = 2). Functional imaging sessions began with four discarded RF excitations to allow for steady state equilibrium. TR’s were 1500 ms for the ER run and 2000 ms for the WoF and resting state runs. Images were presented using ePrime (Psychology Software Tools Inc., Pittsburgh, PA) for the WOF task, and with CIGAL [36] for the ER task.

### Imaging data analysis

Functional data were preprocessed using FEAT 5.92 within FSL 4.0.4 (Oxford Centre for Functional Magnetic Resonance Imaging of the Brain (FMRIB), Oxford University, U.K.). Preprocessing was applied in the following steps: (i) brain extraction for non-brain removal on the T1-weighted image [37], (ii) motion correction using MCFLIRT [38], (iii) spatial smoothing using a Gaussian kernel of FWHM 5 mm, (iv) mean-based intensity normalization of all volumes by the same factor, and (v) high-pass filtering (60 s) [39]. Functional images were co-registered to the brain-extracted T1-weighted image in native space, and the anatomical image was normalized to Montreal Neurological Institute (MNI) standard stereotaxic space. The same transformation matrices used for anatomical-to-standard transformations were then used for functional-to-standard space transformations of co-registered functional images. All registrations were carried out using an intermodal registration tool [37,39]. Voxel-wise temporal autocorrelation was estimated and corrected using FMRIB's Improved Linear Model [40].

For ER and WOF data, onset times of events were used to model a signal response containing a regressor for each response type, which was convolved with a double-gamma function to model the predicted hemodynamic response. A general linear model (GLM) approach generated whole brain images of parameter estimates and variances, representing average signal change from baseline. The same high-pass filtering applied to the functional data was applied to the GLM. Group-wise activation images were calculated by a mixed effects analysis using Bayesian estimation techniques, FMRIB Local Analysis of Mixed Effects [FILM, [41]. Group-level models, with mean-centered BDI scores as regressors, were created by combining contrast maps from individual subjects. Finally, scatterplots of correlations between brain activation and BDI score are presented for illustrative purposes [42].

Analysis of resting state data used the following seed-based connectivity approach: (1) raw functional scans were temporally band-pass filtered (0.1 < *f* < .08); (2) as in [15], the mean signal intensity timecourses from voxels inside a spherical ROI (radius = 10 mm) in the precuneus (MNI coordinates 0, -56, 30) were extracted for all participants in native space; (3) average signal intensity timecourses derived from the previous step were then used as unconvolved regressors in a GLM that included motion parameters, resulting in clusters significantly correlated with the timecourse of the seed region; (4) a group-level model was created by combining contrast maps from individual participants.

Given the importance of protecting against Type II errors in this exploratory pilot study, activation maps were thresholded at Z > 2.3 with a spatial extent of at least ten voxels, consistent with published recommendations [43,44] and our prior MDD studies [25,45-47].

## Results

### In-scanner emotion regulation success

There were no differences in self-reported ER success across the four ER conditions (Neutral-View, Sad-View, Sad-Distract, and Sad-Reappraise), *p’s* > 0.80. There was a significant inverse relation between BDI scores and success ratings only in the Sad-Reappraise condition, *r* = −0.71, *p* < .01, reflecting poorer self-reported ER in those with higher BDI scores.

### Emotion regulation imaging data

#### Sad-reappraise > Sad-view

**Figure 2:**
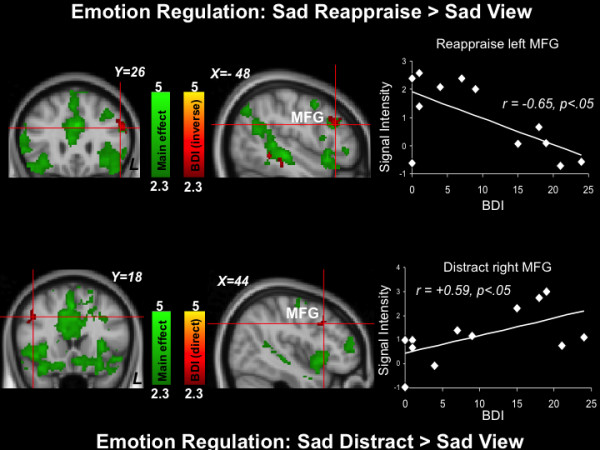
**Emotion regulation results (Top: Sad-Reappraise > Sad View contrast; Bottom: Sad Distract>Sad View contrast).** Green: Clusters significantly activated by the task. Red: Clusters significantly directly (top) and inversely (bottom) related to depression symptom severity. The scatterplots are provided for illustrative purposes and depict correlations between BDI scores with signal intensities of the red left (top) and right (bottom) midfrontal gyrus (MFG) clusters indicated by the crosshairs.

The top of Figure 2 depicts results during Sad-Reappraise > Sad-View (see Table 1). Areas that showed activation included the paracingulate gyrus (Z Max = 4.36), right middle frontal gyrus (Z Max = 3.07), and bilateral temporal gyrus (Z Max = 4.14 for left and 3.81 for right). BDI scores were inversely related to activation magnitudes in the left middle frontal gyrus (Z Max = 2.98) and right orbitofrontal cortex (Z Max = 2.82) (see Table 2).

**Table 1:** Clusters showing significant activation during the Emotion Regulation task [Sad Reappraise > Sad View]

**Region**	**Size (mm**^**3**^**)**	**Z Max**	**MNI Coordinates**		
			**X**	**Y**	**Z**
Frontal Gyrus (Right, Middle)	688	3.07	48	12	48
Frontal Gyrus (Left, Superior)	280	2.83	-18	8	68
Frontal Pole (Left)	472	2.98	-28	-44	-24
Hippocampus (Right)	184	2.54	32	-20	-12
Intracalcarine Cortex (Left)	176	2.56	-18	-68	4
Occipital Cortex (Left, Lateral, Inferior)	896	2.92	-42	-70	-20
Paracingulate Gyrus (Left)	164248	4.36	0	50	12
Putamen (Right)	240	2.92	22	-2	8
Temporal Gyrus (Middle, Posterior)					
Right	8792	3.81	-48	-30	-4
Left	26600	4.14	50	-28	-6

Clusters are > 10 voxels with Z > 2.3.

**Table 2:** Cluster activations inversely associated with BDI scores during the Emotion Regulation task [Sad Reappraise > Sad View]

**Region**	**Size (mm**^**3**^**)**	**Z Max**	**MNI Coordinates**		
			**X**	**Y**	**Z**
Frontal Gyrus (Left, Middle)	744	2.98	-50	24	26
Frontal Orbital Cortex (Right)	296	2.82	42	32	-10
Frontal Pole					
Right	456	3.23	20	72	10
Left	952	3.5	-10	60	30
Parahippocampal Gyrus (Left, Posterior)	168	2.63	-48	-32	-16

Clusters are > 10 voxels with Z > 2.3.

#### Sad-Distract > Sad-View

The bottom of Figure 2 depicts group-average results during Sad-Distract > Sad-View (see also Table 3). Areas that showed activation included the cingulate gyrus (Z Max = 4.54), right supramarginal gyrus (Z Max = 3.63), and right lateral occipital cortex (Z Max = 3.39). BDI scores were directly related to activation magnitudes in the right middle frontal gyrus (Z Max = 2.69), right precentral gyrus (Z Max = 3.09), and left superior temporal gyrus (Z Max = 2.87) (see Table 4).

**Table 3:** Clusters showing significant activation during the Emotion Regulation task [Sad Distract > Sad View].

**Region**	**Size (mm**^**3**^**)**	**Z Max**	**MNI Coordinates**		
			**X**	**Y**	**Z**
Cingulate Gyrus (Right, Posterior)	295512	4.54	0	-48	12
Occipital Cortex (Right, Lateral, Superior)	424	3.39	56	-62	20
Precentral Gyrus (Left)	672	2.78	-56	2	10
Supramarginal Gyrus (Right, Posterior)	3336	3.63	62	-42	24
Temporal Fusiform Cortex (Left, Posterior)	248	2.97	-32	-26	-28
Temporal Gyrus (Right, Superior)	2936	3.21	48	-26	-4

Clusters are > 10 voxels with Z > 2.3.

**Table 4:** Cluster activations directly associated with BDI scores during the Emotion Regulation task [Sad Distract > Sad View]

**Region**	**Size (mm**^**3**^**)**	**Z Max**	**MNI Coordinates**		
			**X**	**Y**	**Z**
Frontal Gyrus (Right, Middle)	288	2.69	44	18	38
Precentral Gyrus (Right)	400	3.09	6	-30	76
Temporal Gyrus (Left, Superior)	192	2.87	-64	-22	0

Clusters are > 10 voxels with Z > 2.3.

### Resting state data

**Figure 3:**
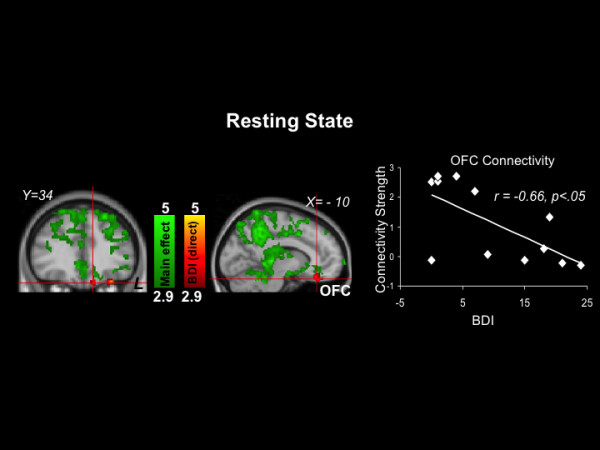
**Resting state results. Green: Clusters with timecourses that significantly correlated with the timecourse of the precuneus seed region.** Red: Clusters significantly directly related to depression symptom severity (there were no clusters with significant inverse associations with depression symptom severity). The scatterplot is provided for illustrative purposes and depicts the correlation of BDI scores with connectivity strength between the precuneus seed and the red orbital frontal cortex (OFC) cluster indicated by the crosshair.

Brain regions that covaried with the precuneus seed are depicted in (Figure 3 and Table 5). Intrinsic functional connectivity was observed between the precuneus seed and left postcentral gyrus (Z Max = 5.19), right orbital frontal cortex (Z Max = 3.61), and bilateral temporal pole (Z Max = 4.38 for right and 3.43 for left). BDI scores directly predicted the strength of functional connectivity between the precuneus seed and left orbitofrontal cortex (Z Max = 4.42), left thalamus (Z Max = 3.58), right occipital cortex (Z Max = 3.96), bilateral parietal lobule (Z Max = 5.81 for right and 3.91 for left), bilateral postcentral gyrus (Z Max = 4.71 for right and 3.89 for left), and bilateral precentral gyrus (Z Max = 4.55 for right and 4.59 for left) (see Table 6).

**Table 5:** Clusters showing significant connectivity with the precuneus seed region during the resting state scan

**Region**	**Size (mm**^**3**^**)**	**Z Max**	**MNI Coordinates**		
			**X**	**Y**	**Z**
Frontal Gyrus, pars opercularis (Left, Inferior)	24	3.79	-58	14	28
Frontal Orbital Cortex (Right)	21	3.61	44	16	-10
Frontal Pole					
Right	157	3.75	20	66	-4
Left	83	3.73	-32	36	-10
Insular Cortex (Left)	32	3.64	-32	14	6
Occipital Cortex (Right, Lateral, Superior)	117	4.64	42	-74	18
Occipital Fusiform Gyrus (Right)	215	4.49	20	-80	-18
Occipital Pole (Left)	29	4.69	-42	-92	4
Planum Temporale (Right)	28	3.57	56	-26	8
Postcentral Gyrus (Left)	59	5.19	-48	-20	54
Temporal Fusiform Cortex (Left, Posterior)	20	4.11	-68	-26	-20
Temporal Gyrus (Right, Middle, Posterior)	158	3.9	60	-20	-12
Temporal Gyrus, temporooccipital part (Right, Inferior)	61	3.79	46	-56	-12
Temporal Pole					
Right	34	4.38	48	6	-26
Left	42	3.43	-44	10	-18

Clusters are > 10 voxels with z > 2.9.

**Table 6:** Clusters directly associated with BDI scores during the resting state scan

**Region**	**Size (mm**^**3**^**)**	**Z Max**	**MNI Coordinates**		
			**X**	**Y**	**Z**
Cingulate Gyrus (Left, Posterior)	37	3.56	-4	-20	42
Frontal Gyrus (Right, Superior)	40	3.96	14	2	66
Frontal Orbital Cortex (Left)	79	4.42	-30	34	-22
Frontal Pole					
Left	73	4.13	-8	40	-26
Right	98	4.27	38	38	8
Lingual Gyrus (Right)	28	3.53	30	-46	-4
Occipital Cortex (Left, Lateral, Inferior)	27	3.55	-38	-74	-2
Occipital Cortex (Lateral, Superior)	25	3.53	42	-74	16
Left	23	3.25	-24	-78	24
Right	51	3.56	42	-58	36
Occipital Cortex (Right, Lateral, Superior)	208	3.96	20	-76	38
Occipital Pole (Right)	29	3.76	12	-90	20
Parietal Lobule (Superior)					
Left	28	3.91	-32	-54	70
Right	781	5.81	26	-46	60
Planum Temporale (Left)	77	4.56	-60	-14	2
Postcentral Gyrus					
Left	46	3.89	-48	-18	34
Right	529	4.71	48	-20	52
Precentral Gyrus					
Left	174	4.59	-58	2	18
Right	285	4.55	30	-10	46
Thalamus (Left)	23	3.58	-12	-32	4

Clusters are > 10 voxels with z > 2.9.

### In-scanner wheel of fortune affective ratings

Average (SD) valence ratings on a scale of 1–6 after non-win trials was 2.87 (1.19) (1 depicted a very sad face and 6 depicted a neutral face). Average (SD) valence after win trials was 4.94 (0.86) (1 depicted a neutral face and 6 depicted a very happy face). BDI scores were not significantly correlated with valence ratings after win trials, *r* = −0.44, *p* = 0.15 or after non-win trials, *r* = −0.03, *p* =0.93.

### Wheel of fortune imaging data

Consistent with published approaches [22,35], analysis for each phase of the WOF task contrasted monetary and control trials.

### Reward selection phase

**Figure 4:**
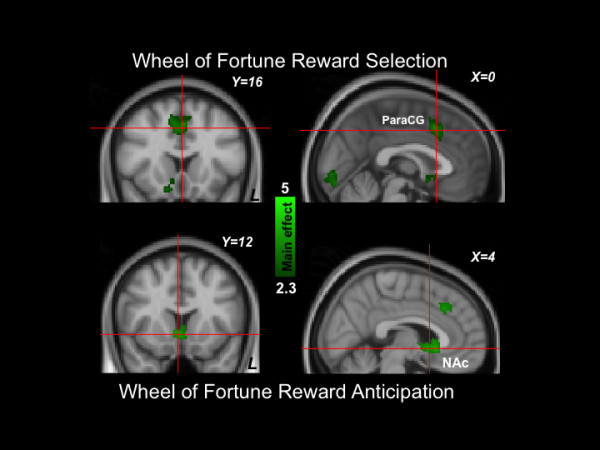
**Wheel of Fortune results (Top: reward selection; Bottom: reward anticipation).** Green: Clusters significantly activated by the task. No regions were significantly related to depression symptom severity. ParaCG: Paracingulate gyrus; NAc: nucleus accumbens.

The top of Figure 4 depicts results during reward selection (see also Table 7). Areas that showed activation included dorsal paracingulate gyrus (Z Max = 3.45), anterior cingulate gyrus (Z Max = 2.97), and right frontal orbital cortex (Z Max = 3.15). BDI scores did not predict activation magnitudes in any region during this phase of the task.

**Table 7:** Clusters showing significant activation during the selection phase of the Wheel of Fortune task

**Region**	**Size (mm**^**3**^**)**	**Z Max**	**MNI Coordinates**		
			**X**	**Y**	**Z**
Cingulate Gyrus (Left, Anterior)	192	2.97	-6	34	18
Frontal Orbital Cortex (Right)	1264	3.15	16	10	-14
Frontal Pole (Right)	272	2.57	40	50	0
Occipital Cortex (Left, Lateral, Inferior)	160	2.99	-42	-72	-18
Occipital Cortex (Right, Lateral, Superior)	25000	3.81	-18	-60	68
Occipital Fusiform Gyrus (Right)	24440	3.75	-22	-88	-14
Paracingulate Gyrus (Right)	5288	3.45	0	16	44
Parietal Lobule (Left, Superior)	272	2.78	-20	-54	54
Subcallosal Cortex (Left)	488	2.78	-4	14	-10
Supramarginal Gyrus (Left Anterior)	616	3	-52	-28	48
Temporal Gyrus, temporooccipital (Right, Middle)	208	2.67	48	-58	-2

Clusters are > 10 voxels with Z > 2.3.

### Reward anticipation phase

The bottom of Figure 4 depicts results during reward anticipation (see also Table 8). Areas that showed activation included bilateral nucleus accumbens (Z Max = 3.44 for right and 2.79 for left,), right putamen (Z Max = 2.67), and right thalamus (Z Max = 2.8). BDI scores did not predict activation magnitudes in any regions during this phase of the task.

**Table 8:** Clusters showing significant activation during the anticipation phase of the Wheel of Fortune task

**Region**	**Size (mm**^**3**^**)**	**Z Max**	**MNI Coordinates**		
			**X**	**Y**	**Z**
Accumbens					
Right	3560	3.44	6	18	-4
Left	496	2.79	-12	8	-12
Lingual Gyrus (Right)	344	2.68	12	-90	-4
Occipital Cortex (Left, Lateral, Inferior)	1160	2.98	-34	-80	2
Occipital Cortex (Lateral, Superior)					
Right	2632	3.54	36	-84	12
Left	536	3	-24	-60	34
Occipital Pole (Left)	416	2.75	-2	-92	-8
Paracingulate Gyrus (Right)	1288	2.99	2	26	38
Parietal Lobule (Superior)					
Right	1296	2.83	18	-58	62
Left	392	2.78	-22	-48	48
Putamen (Right)	104	2.67	16	14	-12
Thalamus (Right)	360	2.8	12	-20	18

Clusters are > 10 voxels with Z > 2.3.

### Reward outcome phase

**Figure 5:**
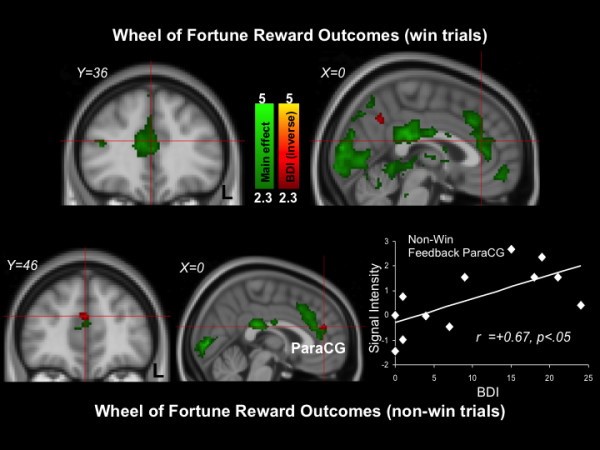
**Wheel of Fortune results (Top: win outcomes; Bottom: non-win outcomes).** Green: Clusters significantly activated by the task. Red: Clusters significantly inversely related to depression symptom severity. The scatterplot is provided for illustrative purposes and depicts correlations between signal intensity of the red paracingulate gyrus (ParaCG) cluster indicated by the crosshair with BDI scores.

The top of Figure 5 depicts results during win outcomes (see also Table 9). Areas that showed activation included the left putamen (Z Max = 3.1), right thalamus (Z Max =2.89), and right amygdala (Z max = 3.02). BDI scores did not predict activation magnitudes in any canonical reward regions during win outcomes. The bottom of Figure 5 depicts average results during non-win outcomes (see also Table 10). Areas that showed activation included the left paracingulate gyrus (Z max = 3.61), left putamen (Z Max = 2.80), and left thalamus (Z Max = 2.96). BDI scores were directly related to activation during non-win outcomes in the left paracingulate gyrus (Z Max = 2.62) and the right caudate nucleus (Z Max = 2.96) (See Table 11).

**Table 9:** Clusters showing significant activation during the outcome phase on win trials of the Wheel of Fortune task

**Region**	**Size (mm**^**3**^**)**	**Z Max**	**MNI Coordinates**		
			**X**	**Y**	**Z**
Amygdala (Right)	280	3.02	10	-4	-16
Central Opercular Cortex (Right)	104	2.68	46	-16	16
Cingulate Gyrus (Left, Anterior)	10160	3.61	-6	40	10
Cingulate Gyrus (Right, Posterior)	7144	4.19	2	-30	24
Frontal Gyrus (Left, Middle)	408	2.94	-50	18	36
Frontal Gyrus (Right, Superior)	88	2.76	24	2	62
Frontal Gyrus, pars triangularis (Left, Inferior)	1400	3.06	-42	34	16
Frontal Orbital Cortex					
Right	664	2.83	18	14	-16
Left	64	2.47	-24	14	-16
Frontal Pole					
Right	144	2.88	34	44	6
Left	480	2.99	-42	40	28
Insular Cortex (Left)	976	3.33	-38	14	-14
Occipital Cortex (Left, Lateral, Superior)	352	2.67	-36	-80	26
Occipital Cortex (Right, Lateral, Inferior)	304	2.77	46	-76	12
Occipital Fusiform Gyrus (Left)	82544	4.49	-20	-88	-8
Parietal Lobule (Right, Superior)	11824	3.67	30	-54	52
Putamen (Left)	2472	3.1	-22	2	8
Thalamus (Right)	704	2.89	20	-16	14

Clusters are >10 voxels with Z > 2.3.

**Table 10:** Clusters showing significant activation during the outcome phase on non-win trials of the Wheel of Fortune task

**Region**	**Size (mm**^**3**^**)**	**Z Max**	**MNI Coordinates**		
			**X**	**Y**	**Z**
Cingulate Gyrus (Posterior)	3128	3.8	0	−30	24
Cuneal Cortex	64	2.46	0	−84	20
Frontal Gyrus (Left, Middle)	1120	3.12	−50	14	38
Frontal Gyrus (Left, Superior)	344	2.9	−8	16	60
Frontal Medial Cortex (Right)	176	2.57	4	54	−8
Frontal Operculum Cortex (Left)	72	2.6	−46	10	0
Frontal Orbital Cortex					
Right	304	2.82	38	18	−16
Left	120	2.92	−24	24	−4
Frontal Pole (Left)	168	2.69	−16	52	40
Hippocampus (Right)	408	2.85	22	−28	−6
Insular Cortex (Left)	1800	3.14	−36	14	−14
Intracalcarine Cortex	27536	4.12	0	−86	2
Lingual Gyrus (Right)	216	2.61	8	−56	−6
Occipital Cortex (Right, Lateral, Superior)	136	2.93	26	−60	34
Occipital Fusiform Gyrus (Right)	104	2.55	30	−68	−18
Paracingulate Gyrus (Left)	8912	3.61	−6	24	34
Parietal Lobule (Left, Superior)	7128	3.25	−32	−46	40
Parietal Lobule (Right)	872	2.86	26	−52	42
Postcentral Gyrus (Left)	216	2.66	−54	−24	42
Putamen (Left)	616	2.8	−22	8	4
Supramarginal Gyrus (Left, Anterior)	1136	3.14	−48	−36	44
Temporal Gyrus (Left, Middle, Posterior)	352	2.88	−58	−34	−4
Temporal Occipital Fusiform Cortex (Right)	104	2.54	36	−56	−8
Temporal Pole (Left)	80	2.62	−54	14	−6
Thalamus (Left)	1944	2.96	−10	−22	4

Clusters are > 10 voxels with Z > 2.3.

**Table 11:** Clusters inversely associated with BDI scores during the outcome phase of the Wheel of Fortune task

**Region**	**Size (mm**^**3**^**)**	**Z Max**	**MNI Coordinates**		
			**X**	**Y**	**Z**
Angular Gyrus (Left)	520	2.73	−38	−56	18
Caudate (Right)	80	2.57	6	10	−2
Cingulate Gyrus (Right, Posterior)	432	2.82	6	−34	44
Frontal Gyrus (Middle)					
Right	248	3.04	28	18	46
Left	152	2.58	−30	26	48
Frontal Gyrus (Superior)					
Right	184	2.65	20	28	44
Left	144	2.56	−6	56	24
Frontal Gyrus, pars opercularis (Right, Inferior)	72	2.53	52	16	28
Frontal Pole (Left)	1008	2.93	−18	56	22
Occipital Cortex (Right, Lateral, Superior)	232	2.6	38	−66	36
Paracingulate Gyrus (Left)	200	2.62	−4	44	22
Postcentral Gyrus (Left)	80	2.65	−48	−16	32
Precentral Gyrus (Left)	736	2.9	−16	−16	68
Precuneous Cortex	64	2.62	0	−58	38
Temporal Gyrus (Left, Middle, Posterior)	288	2.96	−58	−24	−12
Temporal Gyrus, temporooccipital part (Right, Middle)	600	2.9	64	−40	−10

Clusters are > 10 voxels with Z > 2.3.

## Discussion

The goal of the present study was to use fMRI to investigate linkages between patterns of brain activation and connectivity and subclinical levels of depressive symptoms. Emotion regulation, resting state, and reward processing paradigms were used that have been shown to differentiate MDD and control samples and to be related to core features of MDD. During the ER task, participants with higher BDI scores reported significantly poorer self-reported ER success. This pattern is consistent with the findings of Beauregard and colleagues [11], who found that individuals with MDD reported relatively greater difficulty down-regulating feelings of sadness. Consistent with reports from nonclinical samples [6], group-level activation during reappraisal of sad stimuli was evident in the right middle frontal gyrus and bilateral temporal gyrus. Depressive symptom severity was related to activation magnitudes in right middle frontal gyrus during emotion regulation, though the laterality and direction of this finding was contingent on the type of ER strategy: left middle frontal gyrus activation was inversely related to activation during reappraisal, whereas right middle frontal gyrus activation was directly related to activation during distraction. These findings are consistent with neuroimaging data indicating that MDD is characterized by increased right middle frontal activation during reappraisal [6]. More broadly, these results suggest that brain activation during ER is related to the magnitude of subclinical levels of depressive symptoms.

During the resting state scan, intrinsic connectivity was observed between the precuneus seed region and the temporal cortex and anterior cingulate, similar to findings previously reported in nonclinical samples [15]. There was a significant positive correlation between depression symptom severity and the strength of functional connectivity between the precuneus seed region and the thalamus and left orbital frontal cortex. Given that MDD is characterized by increased DMN connectivity both during emotional processing paradigms and at rest [18,19], the present findings suggest that increased DMN connectivity may characterize individuals with increasing levels of subclinical depressive symptoms, possibly reflecting heightened self-referential activities and ruminative cognitions.

During all phases of the reward processing task, participants demonstrated frontostriatal brain activation patterns similar to published reports from nonclinical samples, including dorsal paracingulate cortex activation during reward selection, striatal activation during reward anticipation, and medial prefrontal activation during reward outcomes [25,35]. Depressive symptom severity was not related to activation magnitudes in canonical reward regions during reward selection, reward anticipation, or win outcomes. However, depressive symptom severity was directly related to paracingulate gyrus activation during non-win outcomes. This pattern of findings is noteworthy when considered alongside the results of Pizzagalli and colleagues [23], who found greater differences between MDD and control samples during reward outcomes than during reward anticipation. There is also evidence that pediatric MDD is characterized by altered neural mechanisms of reward anticipation following reward outcomes [48], suggesting that neural responses to reward outcomes may impact future experiences with rewards. More generally, this pattern of results suggests that altered neural correlates of non-win reward outcome may be a more sensitive marker of subclinical depressive symptoms than neural responses during reward selection, reward anticipation or win outcomes.

The present study has a number of methodological limitations that should be considered when interpreting results. The relatively small sample and inclusion of only females suggests the need for replication in larger and more heterogeneous samples. Monthly variability in mood symptoms tied to menstrual phase and use of hormone contraceptives were not assessed, nor was participant handedness ^1^. Finally, brain imaging analyses were conducted at uncorrected thresholds to protect against Type II errors in this pilot study, and future studies with larger samples will allow for replication with corrected statistical thresholds.

## Conclusions

Despite these limitations, this study suggests that subclinical depressive symptom severity is selectively related to regional brain activation patterns in key emotion regulation and reward processing regions and to DMN connectivity implicated in MDD. Although the three domains addressed here (i.e., emotion regulation, resting state connectivity, and reward processing) were assessed via separate tasks, there is clear conceptual overlap between these three domains. For example, prefrontal cortical recruitment is anomalous in MDD during regulation of rewarding stimuli [10] and DMN activity in MDD is abnormal during emotion regulation [18]. In this regard, the present findings may represent a conservative estimate of the effects of subclinical depressive symptoms on neural responses during tasks that simultaneously address these constructs.

Given that individuals with subclinical levels of depressive symptoms are at heightened risk for developing MDD [1,2], these results suggest candidate neural markers that may reflect trait vulnerability to MDD. Because MDD represents a highly prevalent, chronic, and costly disorder, identification of at risk individuals may provide critical windows for preventative interventions that represents a public health priority [49]. Future research using longitudinal and high-risk designs will be needed to more fully evaluate etiologic questions about MDD, MDD risk, and MDD resilience.

## Endnotes

^a^We thank an anonymous reviewer for raising these points.

## Abbreviations

BDI: Beck Depression Inventory-II; BOLD: Blood Oxygen Level Dependent; DMN: Default Mode Network; ER: Emotion Regulation; fMRI: Functional magnetic resonance imaging; GLM: General linear model; MDD: Major Depressive Disorder; MNI: Montreal Neurological Institute; WOF: Wheel of Fortune; SCID: Structured Clinical Interview for DSM-IV Patient Version.

## Competing interests

The authors declare that they have no competing interests.

## Authors' contributions

JNF, MJS, and GSD conceived of and conducted the study and drafted the manuscript. RVK, BF, JM, JB, and CP participated in study design and data analysis and helped to draft the manuscript. All authors read and approved the final manuscript.

## Pre-publication history

The pre-publication history for this paper can be accessed here:

http://www.biomedcentral.com/1471-244X/12/152/prepub
